# Influences of micro-geomorphology on the stoichiometry of C, N and P in Chenier Island soils and plants in the Yellow River Delta, China

**DOI:** 10.1371/journal.pone.0189431

**Published:** 2017-12-13

**Authors:** Fanzhu Qu, Ling Meng, Junbao Yu, Jingtao Liu, Jingkuan Sun, Hongjun Yang, Linshui Dong

**Affiliations:** 1 Shandong Provincial Key Laboratory of Eco-Environmental Science for Yellow River Delta, Binzhou University, Binzhou, Shandong, P. R. China; 2 Institute of coastal ecology, LuDong University, Yantai, Shanodng, P.R. China; Shandong University, CHINA

## Abstract

Studies have indicated that consistent or well-constrained (relatively low variability) carbon:nitrogen:phosphorus (C:N:P) ratios exist in large-scale ecosystems, including both marine and terrestrial ecosystems. Little is known about the C, N and P stoichiometric ratios that exist in the soils and plants of Chenier Island in the Yellow River Delta (YRD). We examined the distribution patterns and relationships of C, N and P stoichiometry in the soils and plants of Chenier Island, as well as the potential influences of the island’s micro-geomorphology. Based on a study of four soil profile categories and *Phragmites australis* and *Suaeda heteroptera* plant tissues, our results showed that micro-geomorphology could leave a distinct imprint on the soil and plant elemental stoichiometry of Chenier Island; significant variation in the atomic C:N:P ratios (R_CNP_) existed in soils and plants, indicating that the R_CNP_ values in both the soil and plants are not well constrained at the Chenier Island scale. R_CN_ and R_CP_ in Chenier Island soils were high, whereas the R_NP_ values were comparatively low, indicating that the ecosystems of Chenier Island are nutrient-limited by N and P. However, the R_NP_ values in *P*. *australis* and *S*. *heteroptera* plant tissues were high, suggesting that the plants of Chenier Island are nutrient-limited by P. Finally, we suggest that soil and plant N:P ratios may be good indicators of the soil and plant nutrient status during soil development and plant growth, which could be a useful reference for restoring the degraded soils of Chenier Island.

## Introduction

All organisms are composed of the same biogenic elements, providing a mechanistic link from atoms to molecules, organisms, and processes that is applicable at scales from the cell to the biosphere [1]. Carbon (C), nitrogen (N) and phosphorus (P) are the most important essential elements that are not found in consistent proportions in all living organisms, and key characteristics of organisms and ecosystems are determined by the dynamics of elemental ratios [2]. Based on work by Fleming (1940), Alfred Redfield (1958) declared the average atomic C:N:P ratio of planktonic biomass to be 106:16:1, a ratio that has since been established as the “Redfield ratio” [3]. The elegant simplicity of this stoichiometric relationship–the Redfield ratio–belies its utility and predictive power, which has prompted ecologists to search for similar patterns and relationships in terrestrial ecosystems and has even inspired a new discipline of ecological stoichiometry, which provides an integrative approach for such analyses of community and ecosystem structure across Earth’s diverse habitats [2,4]. Ecological stoichiometry has already proven to be valuable in examining the relationships between organisms and ecosystem structure and function with the environment and organism C:N:P stoichiometry [5–7]. Understanding ecological stoichiometry is critical in understanding various connections between trophic interactions and nutrient cycling [8–11]. Ecological stoichiometric theories have influenced our knowledge of the first law of thermodynamics, natural selection and evolutionary biology, and the central dogma of molecular biology [12–13], resulting in successful evaluations on different scales, from molecules to organisms and from ecosystems to the biosphere [4–5,14–15]. Meanwhile, to date, few work on C, N, and P stoichiometric characteristics of soils and plants in coastal wetlands has been reported.

Coastal wetlands play an important role in global nutrient cycling because they are the most productive ecosystems on our planet [16]. Soils and plants are two primary components of wetlands. The Chenier Wetland is a special type of coastal wetland, a fragile ecosystem found in one of the most interactive land-ocean regions of the world [17]. The Yellow River is an essential river for China’s very existence and whose basin was the birthplace of ancient Chinese civilization. The Chenier Island of the Yellow River Delta (YRD) is located at the Binzhou Shell Dike Islands and Wetlands National Nature Reserve, which was established in February 2006 by the State Council of China and covers 43 500 ha, 15 500 ha of which are considered core area. *Phragmites australis* (Cav.) Trin. ex Steud. is a cosmopolitan plant found throughout the world that can spread more than 0.05 ha in 2 years [2]. Additionally, *Suaeda heteroptera* Kitag. is a pioneer salt-tolerant plant species that is widely distributed in coastal wetland communities. *P*. *australis* and *S*. *heteroptera* can survive in most coastal wetland habitats, and Chenier Island in the Yellow River Delta is no exception.

Previous studies on Chenier Island focused on either plant physiological characteristics and soil physicochemical properties or plant physiological characteristics and biodiversity [18]. Until now, little research has been carried out on C, N and P stoichiometry of Chenier Island soils and plants in the YRD. Thus, we have chosen this area to carry out our work. Our objectives in this study were to (1) investigate the general C:N, C:P and N:P ratios in Chenier Island soils and plant tissues, (2) verify whether well-constrained C:N:P ratios exist on Chenier Island, and (3) analyze and confirm the influence of micro-geomorphology on the C:N:P ratios of the marshlands on Chenier Island.

## Materials and methods

### Study area

**Fig 1:**
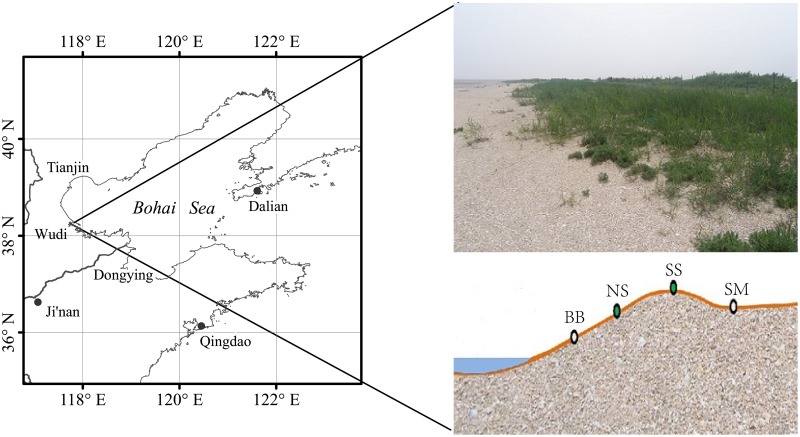
Location of the sampling sites in Chenier Island in the Yellow River Delta, China.

The study area is located on the Binzhou Shell Dike Islands and Wetlands National Nature Reserve, which is the Chenier Wetland ecosystem of the YRD in Wudi County, Shandong Province, China (Fig 1). The county (N37°41′-38°18′, E117°31′-118°12′) lies in the East Asia Monsoon Continental Sub-humid Climate Zone and is classified as a warm temperate zone. It is characterized by a mild climate with four distinct seasons and obvious wet and dry seasons. The annual precipitation in the study region ranges from 534.5–816.1 mm, with nearly 70% of the precipitation falling during summer. The mean annual temperature is 13.6°C. The typical chenier island of this study is well known as one of three ancient shell dikes, in addition to the chenier ridges in southwestern Louisiana and the chenier coast of Suriname [19]. They were formed by coastal sands made from debris from shells. Chenier Island has never been an island, and it is unlikely it will ever become an island. Chenier Island is a chenier, a shell-sand ridge surrounded by marshland and reed swamps. Chenier islands often occur in subparallel sets as elongated, narrow ridges (width: ≤ 400 m; maximum elevation: 2–3 m), standing at or slightly above the maximum high-tide level. The microtopography of Chenier Island is characterized by higher elevations in the middle and southern regions and lower elevation in the northern region due to wave action. The wide spatial distribution of sandflats and marshes is an important characteristic of this coastal wetland. Its soil matrix is composed of shell, sand and mud, with a unit weight of 1.2 g·cm^-3^, coarse particle diameter, large porosity, weak water and fertilizer retention and large daily temperature fluctuations, with rapid increases during the day and decreases at night.

### Sample collection, preparation and chemical analysis

Samples from four site categories were collected on September 5, 2015 to assess the influence of micro-geomorphology on C, N and P stoichiometry in soils and plants of Chenier Island in the YRD. The plots were classified as follows: (1) bare beach (BB, Plot 1: N38°13′34″, E117°57′18″; Plot 2: N38°13′31″, E117°57′18″ and Plot 3: N38°13′27″, E117°57′24″), found at the bottom of the northern beach ridge of Chenier Island with no vegetation cover; (2) north side (NS, Plot 1: N38°13′34″, E117°57′17″; Plot 2: N38°13′30″, E117°57′21″ and Plot 3: N38°13′26″, E117°57′23″), found on the north side of beach ridge with *P*. *australis* and *S*. *heteroptera* as the dominant species cover; (3) south side (SS, Plot 1: N38°13′34″, E117°57′15″; Plot 2: N38°13′30″, E117°57′19″ and Plot 3: N38°13′26″, E117°57′22″), found on the top of beach ridge with *P*. *australis* and *S*. *heteroptera* as the dominant species cover; and (4) salt marsh (SM, Plot 1: N38°13′33″, E117°57′14″; Plot 2: N38°13′25″, E117°57′21″ and Plot 3: N38°13′29″, E117°57′17″), found on the south side of Chenier Island with no salt-marsh vegetation cover. 3 10 m×10 m plots were sampled for each category. In each plot, 6 replicate soil samples were collected using a stainless-steel slide hammer with an inner diameter of 3.5 cm. Each collected sample was sectioned at 10 or 20 cm intervals and then stored in polyethylene plastic bags, which were taken to the laboratory. All soil samples were air-dried, ground using a mortar and pestle, and then sieved through 0.850 mm mesh before chemical analysis. Meanwhile, samples of reed (*P*. *australis*) and seepweed (*S*. *heteroptera*) plant tissues in NS and SS were severed at the ground surface, preserving the belowground portions (root) and the aboveground portions (stem and leaf), which were collected through manual sorting. The samples of each plot were divided into three parts equally in situ from the site that collecting soil. The fresh plant tissues were stored in preservation boxes during shipment to the laboratory. In the laboratory, they were washed with tap water, rinsed with deionized water and then placed in paper bags for fixation (105°C) and drying (80°C).

We state that no specific permissions were required for these locations/activities. We conform that the field studies did not involve endangered or protected species.

### Chemical and statistical analyses

The chemical analyses were carried out at the Shandong Provincial Key Laboratory of Eco-Environmental Science for Yellow River Delta, Binzhou University. Parameters measured include pH, salinity, total carbon (TC), total nitrogen (TN) and the total phosphorous (TP), described in detail by Qu *et al*. [2]. The total C, N and P concentrations (mg/kg) were transformed to units of mmol/kg, so the C:N, C:P and N:P ratios for each soil type and plant sample were calculated as molar ratios (atomic ratio) [20], rather than mass ratios.

The NPAR1WAY procedure with a Kruskal-Wallis test of significance was applied for data comparison using SAS version 8.1 (SAS Inc., Cary, NC). The SPSS version 16.0 (SPSS Inc., NY, USA) was used to calculate Pearson correlation coefficients of C, N and P concentrations and ratios within groups.

## Results

### General characteristics of Chenier Island soils

The mean pH value of ~8.72 was strongly alkaline and observed in all four site categories in the Chenier Island soil profiles (Table 1). The mean pH of the soil significantly differed between SS and the other three sites (*p* = 0.002/*p* = 0.018/*p* < 0.001), which were ranked as SS (mean, 8.92) >> NS (mean, 8.73) > BB (mean, 8.68) >> SM (mean, 8.51), whereas the difference between NS and BB was not significant. The mean salinity was ranked as SM (mean, 6.28 ‰) >> BB (mean, 3.69 ‰) >> NS (mean, 1.12 ‰) > SS (mean, 1.04 ‰), with significant differences (*p* < 0.001) between SM and BB, as well as between BB and NS; however, no significant difference was detected between NS and SS. The soil TC averaged 7.83 g/kg, 5.57 g/kg, 6.40 g/kg and 6.43 g/kg in BB, NS, SS and SM, respectively (Table 1). The mean content of TC in BB was significantly higher than at the other sites, with no significant differences among NS, SS and SM. The soil TN in BB, NS, SS and SM averaged 96.0 mg/kg, 247.0 mg/kg, 465.2 mg/kg, and 213.4 mg/kg, respectively, and the mean TP values were 189.0 mg/kg, 313.6 mg/kg, 351.5 mg/kg, and 239.2 mg/kg, respectively (Table 1). The mean content of TN was ranked as SS >> NS > SM > BB, with no significant differences among NS, SM and BB; however, the mean content of TP was ranked as SS > NS >> SM > BB, with no significant differences between SS and NS or SM and BB. The mean content of TP in SS was the highest of the four sites, but there was no significant difference in TP between NS and SM. On average, the mean content of TN and TP from the beach ridge with vegetative cover on Chenier Island was higher than those of the bare beach and salt marsh.

**Table 1:** Basic soil characteristics of the study are in Chenier Island.

Sites	Altitude (m)	pH	Salinity (‰)	TC (%)	TN (mg/kg)	TP (mg/kg)
BB	0.8	8.68 ± 0.17 ^bc^	3.69 ± 1.26 ^b^	7.83 ± 1.94 ^a^	96.0 ± 39.1 ^b^	189.0 ± 56.7 ^b^
NS	1.5	8.73 ± 0.14 ^b^	1.12 ± 0.40 ^c^	5.57 ± 1.82 ^b^	247.0 ± 189.3 ^b^	313.6 ± 95.3 ^a^
SS	2.0	8.92 ± 0.20 ^a^	1.04 ± 0.31 ^c^	6.40 ± 1.36 ^b^	465.2 ± 361.3 ^a^	351.5 ± 161.1 ^a^
SM	1.8	8.51 ± 0.25 ^c^	6.28 ± 2.60 ^a^	6.43± 2.05 ^b^	213.4 ± 139.5 ^b^	239.2 ± 56.2 ^b^
Mean	1.5	8.72 ± 0.23	3.03 ± 2.60	6.73 ± 1.83	249.1 ± 245.9	268.1 ± 116.0

Values are the weighted means ± SE.

a, b and c represent significant differences between the means for groups, while the means for groups in homogeneous subsets are displayed as ab and bc (*p* = 0.05, n = 15).

### Correlation of C, N and P in Chenier Island soil

The biogeochemical cycles of C, N and P in terrestrial and marine ecosystems are inextricably linked to one another [21]. However, correlation analysis suggested that there were not significant positive correlations between TC and TN/TP in the Chenier Island soils in the YRD (Table 2). Meanwhile, correlation analysis suggested that there was a significant negative correlation between TC and TP in BB, NS and SM and a significant positive correlation between TN and TP in NS and SS in the beach ridge with vegetation cover in the YRD. These differences might reflect the different nutrient sources; C was created by coastal sand that formed from the debris of shells, and N and P largely formed from the soil parent material and soil microorganisms. Though there was not a significant correlation between R_CN_ and R_CP_ in these four sites, with the exception of SS (*r* = 0.750), a negative correlation between R_CN_ and R_NP_ was observed (*r* = -0.570).

**Table 2:** Correlation matrix for C, N and P concentrations and ratios in Chenier Island soils (n = 60) in the YRD.

Sites		Pearson correlation					
		C	N	P	R_CN_	R_CP_	R_NP_
BB	C	1.00					
	N	.255	1.00				
	P	-.787**	.158	1.00			
	R_CN_	.011	-.796**	-.352.	1.00		
	R_CP_	.917**	-.012	-.909**	.173	1.00	
	R_NP_	.695**	.778**	-.454**	-.558*	.573*	1.00
NS	C	1.00					
	N	-.155	1.00				
	P	-.716**	.712**	1.00			
	R_CN_	.406	-.844**	-.727**	1.00		
	R_CP_	.946**	-.308	-.787**	.490	1.00	
	R_NP_	.282	.870**	.294	-.693**	.140	1.00
SS	C	1.00					
	N	.491	1.00				
	P	-.343	.686**	1.00			
	R_CN_	.573*	-.860**	-.623**	1.00		
	R_CP_	.736**	-.782**	-.814**	.750**	1.00	
	R_NP_	-.374	.891	.325	-.830**	.508	1.00
SM	C	1.00					
	N	.256	1.00				
	P	-.858**	.196	1.00			
	R_CN_	.262	-.717**	-.600*	1.00		
	R_CP_	.902**	.000	-.947**	.438	1.00	
	R_NP_	.581*	.902**	-.209	-.529*	.396	1.00
ALL	C	1.00					
	N	.214	1.00				
	P	-.571**	.706**	1.00			
	R_CN_	.355**	-.580**	-.548**	1.00		
	R_CP_	.889**	-.413**	-.742**	.537**	1.00	
	R_NP_	.092	.871**	.355**	-.570**	-.101	1.00

*Significant at *p*< 0.05.

**Significant at *p* < 0.01.

### Distribution patterns of C, N and P stoichiometry in Chenier Island soils

R_CN_ and R_CP_ in BB averaged 1147.80 and 1198.88, respectively, values that were significantly higher than those in NS, SS and SM. The differences between R_CN_ and R_CP_ among NS, SS and SM were not significant (Table 3). The highest R_NP_ mean value (2.73) appeared in soils from SS and the lowest R_NP_ mean value (1.18) appeared in soils from BB. R_NP_ was significantly different between SS and SM/NS (*p* = 0.074/*p* = 0.008) and between SM and BB (*p* = 0.034) but was not significantly different between NS and BB (*p* = 0.034). Soil C:N:P ratios in BB, NS, SS and SM were 1198.9:1.2:1, 546.1:1.6:1, 547.0:2.7:1 and 780.3:1.1:1, respectively.

**Table 3:** Summary of soil C, N and P stoichiometry in Chenier Island in the Yellow River Delta.

Sites	Sample numbers	R_CN_	R_CP_	R_NP_	R_CNP_
BB	15	1147.8 ± 649.1 ^a^	1198.9 ± 512.7 ^a^	1.18 ± 0.5 ^c^	1198.9:1.2:1
NS	15	471.6 ± 345.7 ^b^	561.1 ± 462.2 ^b^	1.66 ± 1.0 ^bc^	561.1:1.6:1
SS	15	274.1 ± 182.1 ^b^	547.0 ± 222.7 ^b^	2.73 ± 1.4 ^a^	547.0:2.7:1
SM	15	443.2 ± 209.4 ^b^	780.3 ± 418.8 ^b^	2.02 ± 1.1 ^b^	780.3:2.0:1
Mean	60	584.2 ± 510.0	771.8 ± 486.8	1.90 ± 1.2	771.8:1.2:1

a, b and c represent significant differences between the means for groups, while the means for groups in homogeneous subsets are displayed as ab and bc (*p* = 0.05, n = 15).

**Fig 2:**
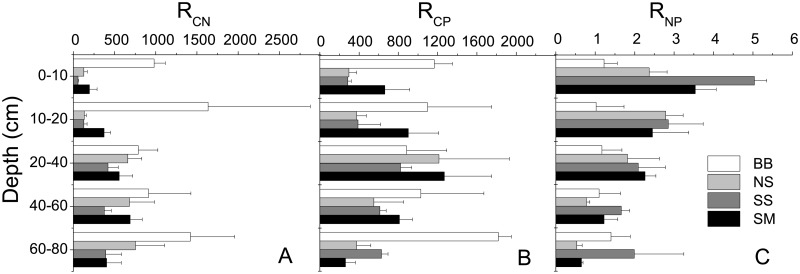
The distribution of R_CN_ (A), R_CP_ (B) and R_NP_ (C) in soil profiles in BB, NS, SS and SM.

The R_CN_, R_CP_ and R_NP_ distributions in the soil profile varied in Chenier Island in the YRD with the influence of micro-geomorphology (Fig 2). In the BB profile, R_CN_ varied with no obvious distribution pattern from the surface to bottom layers, though the R_CN_ values of bottom layers were slightly higher than those of surface layers. The highest R_CN_ value was found in the 10–20 cm layer.—In NS, SS and SM profiles, R_CN_ was lowest in the topsoil and increased with depth, changing slightly at depths of 20–80 cm. The highest R_CP_ value in the BB profile was found at depths of 60–80 cm, with minimal variation at depths between 0–60 cm. Meanwhile, the highest R_CP_ values in the NS, SS and SM profiles were found at depths of 20–40 cm; values were lower in the both the surface and bottom layers. In the BB profile, R_NP_ changed little, with no obvious distribution from surface to bottom. R_NP_ in the NS, SS and SM profiles significantly changed with the same distribution patterns: highest in the topsoil and decreasing with depth.

### C, N and P stoichiometry in different plants of Chenier Island

TC, TN and TP in *P*. *australis* and *S*. *heteroptera* from Chenier Island are presented in Table 4. The mean content of TC and TN in *P*. *australis* tissues (root, stem and leaf) of NS was lower than SS, whereas TP was quite the reverse. The mean content of TC in *S*. *heteroptera* root of NS was higher than those of SS, with no significant differences in stem and leaf between NS and SS. The mean content of TN and TP in *S*. *heteroptera* stem and leaf in NS was lower than those of SS. R_CN_, R_CP_ and R_NP_ in *P*. *australis* and *S*. *heteroptera* from the beach ridge in Chenier Island are presented in Table 5. R_CN_, R_CP_ and R_NP_ averaged 72.19, 1981.24 and 32.98, respectively, in *P*. *australis* of NS and 43.24, 2888.64 and 67.09, respectively, in *P*. *australis* of SS, while R_CN_, R_CP_ and R_NP_ averaged 70.53, 2118.60 and 29.95, respectively, in *S*. *heteroptera* of NS and 35.86, 927.36 and 25.19 in *S*. *heteroptera* of SS. There were significant differences in R_CN_, R_CP_ and R_NP_ in the plant tissues between the north side and the top of the ridge (*F* = 31.88, *p* < 0.001). R_CN_ in the two species of NS were higher than those of SS, while R_CP_ and R_NP_ in *P*. *australis* of SS were higher than those of NS; in contrast, those of *S*. *heteroptera* in SS were lower than those in NS. R_CNP_ averaged 1981:33.0:1 and 2889:67.1:1, respectively, for *P*. *australis* of NS and SS and 2119:30.0:1 and 927:25.2:1, respectively, for *S*. *heteroptera* in NS and SS.

**Table 4:** TC, TN and TP in different plant tissues of *P*. *australis* and *S*. *heteroptera* from Chenier Island.

Plants	Sites	Sample numbers	TC(%)			TN(mg/kg)			TP(mg/kg)		
			Root	Stem	Leaf	Root	Stem	Leaf	Root	Stem	Leaf
*P*. *australis*	NS	18	42.7 ± 3.2	46.3 ± 4.7	45.4 ± 3.8	7930.7 ± 805.4	4302.5 ± 384.0	18853.9 ± 1625.9	472.7 ± 65.1	494.7 ± 45.1	977.9 ± 85.2
	SS	18	46.2 ± 4.0	46.6 ± 4.2	48.3 ± 3.9	11222.3 ± 963.7	9780.3 ± 864.1	21563.4 ± 1847.2	328.6 ± 46.2	362.8 ± 40.7	720.3 ± 52.8
*S*. *heteroptera*	NS	18	44.2 ± 3.4	43.7 ± 4.5	38.6 ± 3.5	5607.8 ± 471.3	6439.0 ± 598.2	11109.6 ± 982.8	406.7 ± 38.3	478.7 ± 48/2	828.6 ± 46.2
	SS	18	30.1 ± 3.6	45.2 ± 3.8	38.8 ± 3.2	10024.1 ± 889.2	10456.3 ± 924.2	20376.2 ± 1489.2	777.9 ± 57.4	880.5 ± 58.2	2163.1 ± 174.6

**Table 5:** Summary of plant C, N and P stoichiometry in the beach ridge in Chenier Island.

Plants	Sites	Sample numbers	R_CN_	R_CP_	R_NP_	R_CNP_
*P*. *australis*	NS	18	72.19 ± 49.4	1981.24 ± 680.1	32.98 ± 12.2	1981:33.0:1
	SS	18	43.24 ±15.3	2888.64 ± 1018.0	67.09 ± 8.0	2889:67.1:1
*S*. *heteroptera*	NS	18	70.53 ± 26.8	2118.60 ± 826.1	29.95 ± 0.5	2119:30.0:1
	SS	18	35.86 ± 14.1	927.36 ± 434.6	25.19 ± 3.9	927:25.2:1

**Fig 3:**
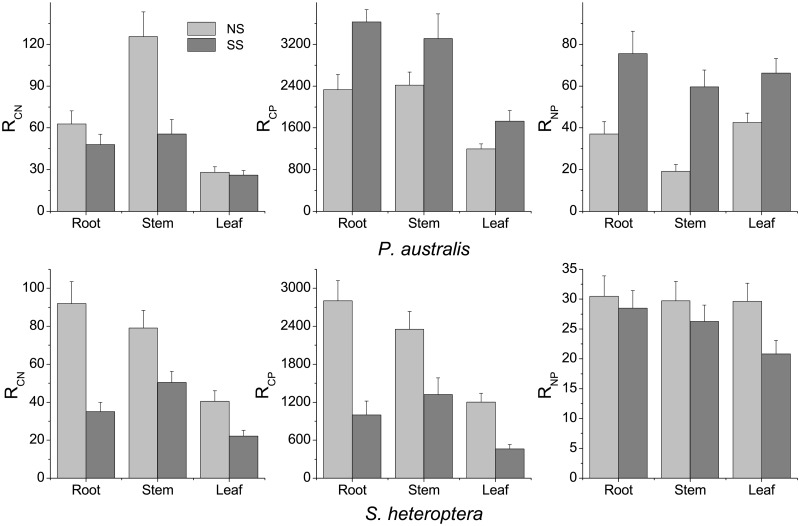
The R_CN_, R_CP_ and R_NP_ in different plant tissues of *P*. *australis* and *S*. *heteroptera* from Chenier Island.

R_CN_, R_CP_ and R_NP_ in the plant tissues (root, stem and leaf) of *P*. *australis* and *S*. *heteroptera* in the beach ridge varied with the influence of micro-geomorphology (Fig 3). There were significant differences in the R_CN_ values of root and stem of *P*. *australis* tissues in NS and SS (*p* < 0.05). There were significant differences in the R_CP_ and R_NP_ values of root, stem and leaf of *P*. *australis* tissues between NS and SS (*p* < 0.05/*p* < 0.01), as well as significant differences in the R_CN_ and R_CP_ values of root, stem and leaf of *S*. *heteroptera* tissues between NS and SS (*p* < 0.01/*p* < 0.01). R_CN_ values of root and stem of *P*. *australis* and *S*. *heteroptera* tissues in NS were significantly higher than those of *P*. *australis* and *S*. *heteroptera* tissues in SS (*p* < 0.01). The R_CP_ and R_NP_ values of root, stem and leaf of *P*. *australis* tissues in NS tended to be lower than those of *P*. *australis* in SS. In contrast, R_CP_ and R_NP_ values of root, stem and leaf of *S*. *heteroptera* tissues in NS were significantly higher than those of *S*. *heteroptera* tissues in SS (*p* < 0.01).

## Discussion

In 1958, Alfred Redfield reported that marine plankton contain C, N, and P in an atomic ratio of 106:16:1, which demonstrated the power of elemental stoichiometry. Since the work done by Alfred Redfield, the use of elemental ratios has laid the foundations for the twentieth-century advances in our understanding of marine biogeochemistry [22]. Deviations from this ratio are now opening an avenue of future exploration for terrestrial ecosystems [23–24]. Based on work by Cleveland and Liptzin (2007), however, there was significant variation in the soil and microbial elemental ratios between vegetation types (i.e., forest versus grassland); the similarities in soil and microbial elemental ratios among sites and across large scales were more apparent than the differences in most cases [4]. On average, the atomic C:N:P ratios in both soil (186:13:1) and soil microbial biomass (60:7:1), which differs significantly from the Redfield ratio, were well-constrained. Based on an inventory data set of 2384 soil profiles from sites in China, Tian et al. (2010) explored the entire soil depth (as deep as 250 cm for some soil profiles), and calculated a C:N:P ratio of ~ 60:5:1, which was not well constrained, though a well-constrained C:N:P ratio of 134:9:1 was found for the 0–10 cm organic-rich soil [25]. To explore global patterns of leaf N and P and their ratio (N:P) in relation to broad-scale variability in geography, temperature, and other climatic factors, Reich and Oleksyn (2004) used a large data set, consisting of 5,087 observations of leaf N and P for 1,280 plant species at 452 sites. They found that plant foliar N:P ratios increased from high to low latitudes, coinciding with biogeographical gradients of soil substrate age and climate [26]. Related studies demonstrated that elemental ratios in terrestrial systems that were characterized by high biological diversity, structural complexity and spatial heterogeneity showed high spatial heterogeneity and large variations between different climatic zones, while parallels seemed to exist in ecosystems that have the most active organism–environment interaction worldwide [25].

Geomorphology plays an important role in chemical cycles in coastal wetland ecosystems, while micro-geomorphology has similar effects on regional ecosystems [27]. Hydraulic and hydrologic regimes (such as changes in water and salt distribution) that depend on micro-geomorphology may greatly affect nutrient spatial distributions [28–29]. Soil salinity, known to influence species composition, growth rates and ecosystem productivity, is an important physiological stress factor for water uptake of coastal vegetation [30–31]. Thus, micro-geomorphology can leave a distinct imprint on soil and plant C, N and P concentrations and ratios. In different micro-geomorphic units of Chenier Island, the C, N and P stoichiometry changed significantly in low-lying sites (BB and NS). R_CN_ and R_CP_ in the bare beach were all higher than those on the beach ridge (NS and SS) and in the salt marsh, while R_NP_ on the south side of the beach ridge (SS and SM) on Chenier Island was higher than those on the north side of beach ridge (NS and BB). The widespread use of fertilizers results in nutrient loading, causing an increase in the R_NP_ on the side nearest the sea. In some aquatic sites, the N:P ratio of soils can be reduced by the high solubility of N. The R_CN_ values for *P*. *australis* on the top of beach ridge (SS) were higher than those on the north side of beach ridge (NS), but R_CP_ and R_NP_ were lower. R_CN_, R_CP_ and R_NP_ for *S*. *heteroptera* on the top of beach ridge (SS) were all lower than those on the north side of beach ridge (NS). The R_NP_ value of the plants suggested the impact of P as a limiting factor in the coastal ecosystems is increasing.

In conclusion, our results confirmed that micro-geomorphology plays a major role in constraining elemental concentrations and ratios in soils and in different tissues of *P*. *australis* and *S*. *heteroptera* on Chenier Island in the YRD. The R_CNP_ values in Chenier Island soils and plants were not well-constrained, as micro-geomorphology had a serious impact on nutrient stoichiometry in soils and plants, primarily because the coastal Chenier Wetland is subjected to dramatic land-ocean interactions. Variation in the micro-geomorphology in small regions had a serious impact on nutrient stoichiometry in soils and the living plants and biogenic elemental cycling over large spatial scales. If we wish to investigate whether the soil C:N:P ratio in organic-rich topsoil could be a good indicator of biogeochemical cycles and nutrient limitations in coastal wetland ecosystems, more spatial, temporal and site-specific studies should be conducted in the future.
